# Diel Patterns of Variable Fluorescence and Carbon Fixation of Picocyanobacteria *Prochlorococcus*-Dominated Phytoplankton in the South China Sea Basin

**DOI:** 10.3389/fmicb.2018.01589

**Published:** 2018-08-02

**Authors:** Yuyuan Xie, Edward A. Laws, Lei Yang, Bangqin Huang

**Affiliations:** ^1^State Key Laboratory of Marine Environmental Science, Xiamen University, Xiamen, China; ^2^Department of Environmental Sciences, Louisiana State University, Baton Rouge, LA, United States; ^3^Department of Environmental Sciences, Xiamen University, Xiamen, China

**Keywords:** diel variation, variable fluorescence, primary production, photosynthetic parameters, *Prochlorococcus*, South China Sea basin, nutrient limitation, photosynthetic energetic stoichiometry

## Abstract

The various photosynthetic apparatus and light utilization strategies of phytoplankton are among the critical factors that regulate the distribution of phytoplankton and primary productivity in the ocean. Active chlorophyll fluorescence has been a powerful technique for assessing the nutritional status of phytoplankton by studying the dynamics of photosynthesis. Further studies of the energetic stoichiometry between light absorption and carbon fixation have enhanced understanding of the ways phytoplankton adapt to their niches. To explore the ecophysiology of a *Prochlorococcus*-dominated phytoplankton assemblage, we conducted studies of the diel patterns of variable fluorescence and carbon fixation by phytoplankton in the oligotrophic South China Sea (SCS) basin in June 2017. We found that phytoplankton photosynthetic performance at stations SEATS and SS1 were characterized by a nocturnal decrease, dawn maximum, and midday decrease of the maximum quantum yield of PSII (${\text{F}}_{\text{v}}(\prime )$/${\text{F}}_{\text{m}}(\prime )$, which has been denoted as both F_v_/F_m_ and ${\text{F}}_{\text{v}}^{\prime }$/${\text{F}}_{\text{m}}^{\prime }$) in the nutrient-depleted surface layer. That these diel patterns of ${\text{F}}_{\text{v}}(\prime )$/${\text{F}}_{\text{m}}(\prime )$ were similar to those in the tropical Pacific Ocean suggests macro-nutrient and potentially micro-nutrient stress. However, the fact that variations were larger in the central basin than at the basin's edge implied variability in the degree of nutrient limitation in the basin. The estimated molar ratio of gross O_2_ production to net production of carbon (GOP:NPC) of 4.9:1 was similar to ratios reported across the world's oceans. The narrow range of the GOP:NPC ratios is consistent with the assumption that there is a common strategy for photosynthetic energy allocation by phytoplankton. That photo-inactivated photosystems or nonphotochemical quenching rather than GOP accounted for most of the radiation absorbed by phytoplankton explains why the maximum quantum yield of carbon fixation was rather low in the oligotrophic SCS.

## Introduction

Phytoplankton are the foundation of marine pelagic ecosystems because they are by far the major primary producers in the ocean (Falkowski et al., 1998). From eutrophic coastal areas to the oligotrophic open ocean, phytoplankton communities show marked gradients of diversity and productivity, and their photosynthetic apparatus has evolved to adapt to different niches (Strzepek and Harrison, 2004; Biller et al., 2015; Xia et al., 2017). Active chlorophyll *a* fluorescence techniques like Fast Repetition Rate fluorometry (FRRf) are acknowledged to be powerful techniques for assessing photosynthetic performance, the different components of nonphotochemical quenching (NPQ), and photoinactivation (Müller, 2001; Campbell and Tyystjärvi, 2012). By providing a mechanism to examine photosynthetic dynamics, this technique can provide insights into the light utilization strategies of different phytoplankton (Behrenfeld and Kolber, 1999; Six et al., 2007; Li et al., 2016; Murphy et al., 2017). The FRRf-derived parameter F_v_/F_m_ represents an estimate of the maximum quantum yield of photochemistry (Kolber and Falkowski, 1993). Researchers usually interpret a decline of F_v_/F_m_ to be an indication that phytoplankton are stressed. Interpretation of F_v_/F_m_ from field samples, however, is confounded by the fact that F_v_/F_m_ varies across taxa, and the magnitude of this taxonomic variability is comparable to the changes induced by nutrient limitation. For chlorophytes and diatoms, F_v_/F_m_ can be as high as 0.65–0.70 under nutrient-replete conditions, whereas the typical F_v_/F_m_ of cyanobacteria is 0.1–0.4 (Suggett et al., 2009b). Thus, the spatial pattern of F_v_/F_m_ is insufficient to assess the physiological state of phytoplankton in natural environments. Additional assays, such as controlled nutrient addition experiments, are therefore usually conducted. In the case of iron fertilization experiments, a significant increase of F_v_/F_m_ is observed upon relief of iron stress (Behrenfeld et al., 2006). However, nutrient addition experiments are labor intensive and cannot be routinely carried out. In contrast, Behrenfeld and Kolber (1999) have demonstrated that autonomous active chlorophyll *a* fluorescence measurements along a cruise track can record diel changes of F_v_/F_m_ that can be used to assess the nutritional state of a phytoplankton assemblage (Behrenfeld and Milligan, 2013). Analysis of such data has provided a synoptic picture of eco-physiological regimes in the tropical Pacific Ocean (Behrenfeld et al., 2006). A complementary examination of the dynamics of phytoplankton carbon fixation can also be revealing. The study by Schuback et al. (2016) has already demonstrated a diurnal pattern of carbon fixation that differed from that of active chlorophyll *a* fluorescence in the subarctic Pacific. Through careful analysis of the dynamics of active chlorophyll *a* fluorescence and carbon fixation of phytoplankton, it is possible to determine the photosynthetic efficiency between light absorption and carbon fixation and to relate that efficiency to the light utilization strategy of the phytoplankton.

*Prochlorococcus* is a class of phytoplankton that is widespread in the tropical and subtropical oceans (Partensky et al., 1999a; Bouman et al., 2006; Johnson et al., 2006). *Prochlorococcus* has been categorized into several high-light (HL) and low-light (LL) ecotypes on the basis of light niches (Biller et al., 2015). Unlike its relative *Synechococcus, Prochlorococcus* does not possess phycobilisomes but has a specific light-harvesting apparatus that is composed of chlorophyll *a/b*-binding proteins encoded by the *pcb* genes (Bibby et al., 2003). Although they are close relatives, *Prochlorococcus* and *Synechococcus* occupy different light niches (Ting et al., 2002; Xiao et al., 2018); in general, *Prochlorococcus* grows at greater depths than *Synechococcus*; but more importantly, *Prochlorococcus* thrives in oligotrophic oceans, where its biomass in terms of carbon is 22 times that of *Synechococcus*, whereas *Synechococcus* is more abundant in nutrient-enriched environments (Partensky et al., 1999a). It is reasonable to hypothesize that *Prochlorococcus* has a trade-off between tolerance to low nutrient and light utilization efficiency. The results of laboratory experiments have shown that, although *Prochlorococcus* and *Synechococcus* have similar responses to excitation pressure in terms of photoinactivation, *Prochlorococcus* cannot maintain photosynthesis at a stable rate under high-light stress conditions, because *Prochlorococcus* invests substantially less energy in repairing damaged photosystems under high-light stress (Bruyant et al., 2005; Six et al., 2007; Mella-Flores et al., 2012; Murphy et al., 2017). *Prochlorococcus* is able to outcompete other phytoplankton for nutrients under oligotrophic conditions, but this capability has been associated with its being less able to deal with high-light stress. However, this idea has not yet been tested in the field.

A cruise during June 2017 in the South China Sea (SCS) basin provided an opportunity for us to conduct high-frequency diurnal cycle measurements at a single station. The SCS is a semi-enclosed marginal sea with a seasonal circulation system driven by monsoon winds (Hu et al., 2000). Previous studies have shown that *Prochlorococcus* is the dominant phytoplankter in the SCS basin during the summer (Xiao et al., 2018) and have revealed maximum quantum yields of carbon fixation in the basin area that are very low, down to <0.01 mol C (mol photons)^−1^ (Xie et al., 2015). In this study, we investigated the diel patterns of variable fluorescence and carbon fixation in this marginal sea; we also estimated the different “photosynthetic currencies” (Suggett et al., 2009a) and absorption coefficients (see the Methods section Estimates of “photosynthetic currencies” and absorption coefficients) to determine the energetic stoichiometry of photosynthesis. The nutritional status of the phytoplankton assemblage and the eco-physiological basis for the low maximum quantum yield of carbon fixation were the foci of this research.

## Methods

### Diurnal cycle measurements

**Figure 1:**
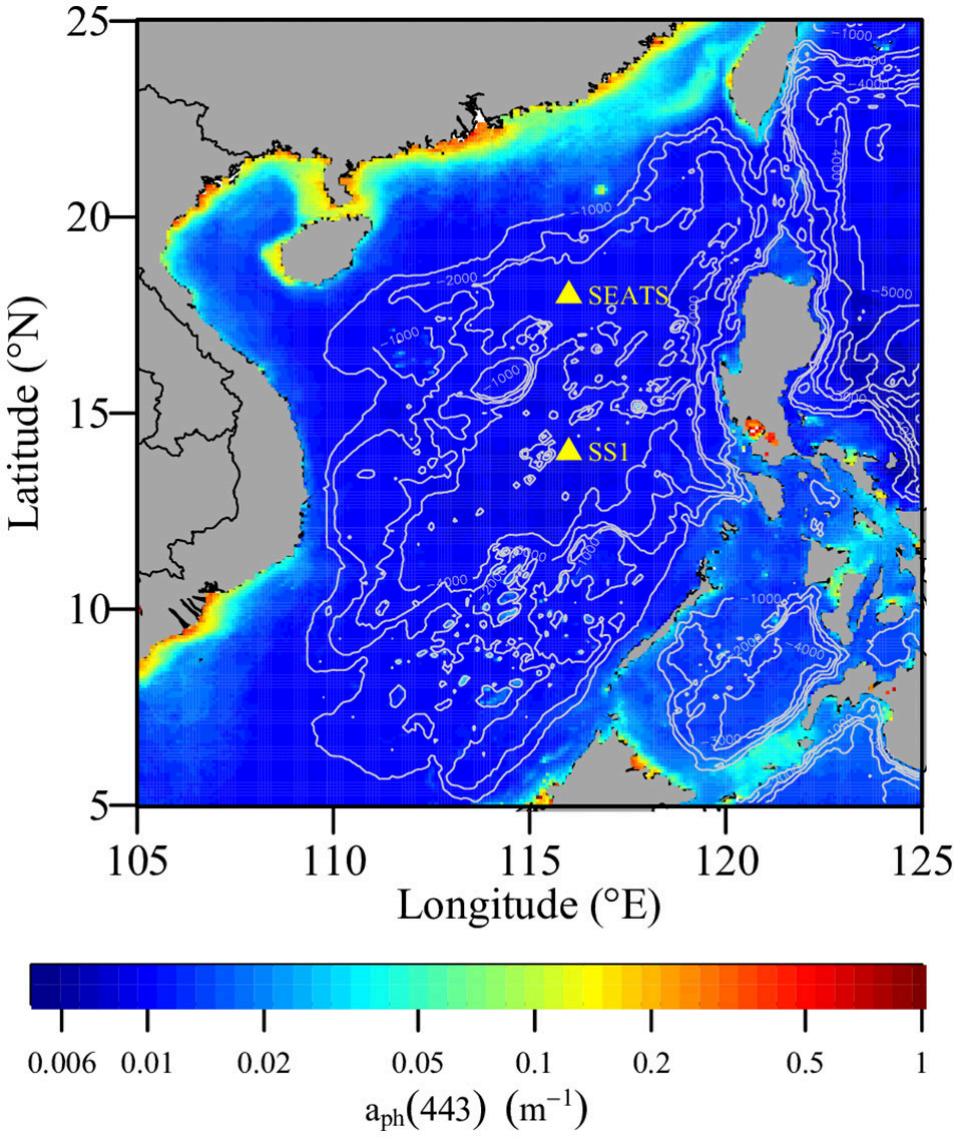
Sampling stations in the South China Sea in June 2017. In the background, the contour lines are water depths, and the color contour is climatologic phytoplankton absorption coefficient at 443 nm in June that is MODIS-Aqua QAA-a_ph_(443) downloaded at https://oceancolor.gsfc.nasa.gov/cgi/l3.

Generally, the SCS basin is oligotrophic (Ning et al., 2004). During cruise KK1702 of the R/V *Tan Kah Kee*, we conducted diurnal cycle measurements at the South East Asia Time-Series Station (SEATS) (116°E, 18°N) between 7 June 2017 and 10 June 2017 at station SS1 (116°E, 14°N) between 14 June 2017 and 17 June 2017 (Figure 1). Table 1 summarizes the diel patterns of physical, chemical, and biological processes in the upper ocean that were investigated.

**Table 1:** Details of diurnal cycle measurements at station SEATS and SS1 in June 2017.

**Item**	**Parameters**	**Depths**	**Frequency**	**Period**
CTD	**Temperature Salinity**	0–300 m.	At an interval of 1.5 h or 3 h at station SEATS; at an interval of 1.5 h at station SS1.	June 7th, 9:20–June 10th, 10:10 at station SEATS; June 14th, 8:30–June 17th, 10:00 at station SS1.
Nutrients	**Nitrate**+**nitrite Phosphate Silicate**	5, 15, 25, 30, 40, 50, 75, 100, 125, 150, 175, 200, 250, 300 m.	At an interval of 3 h.	
FRRf	${\text{F}}_{\text{v}}(\prime )$/${\text{F}}_{\text{m}}(\prime )$$\sigma_{\text{PSII}}(\prime )$	0–100 m.	At varied intervals at station SEATS; at an interval of 1.5 h or 3 h at station SS1.	
NPP	NPP	At the surface and the depths with 70, 50, 23, 12, 3, 1, 0.3% of surface PAR.		June 8th, 9:30, at station SEATS, 24 h incubation; June 15th, 7:30, at station SS1, 24 h incubation.
P-I curve	$P_{m}^{B}$ α β	3, 8, 15, 76, 102 m.		June 16th, 7:30, at station SS1, 1 h incubation; June 16th, 13:30, at station SS1, 1 h incubation.
Chl*a*	Chl*a*	Along with NPP and P-I curve.		Along with NPP and P-I curve.
Pigments	**Pigments Classes**	5, 25, 50, 75, 100, 150 m.	At an interval of 3 h.	June 7th, 7:00–June 8th, 7:50 at station SEATS; June 14th, 8:30–June 17th, 10:00 at station SS1.

*Parameters in bold mean that only part of these data is presented in this study (see the main text)*.

### FRRf measurements

Vertical profiles (0–100 m) of PSII fluorescence were measured with a FastOcean APD (Ambient Plus Dark) fluorometer (Chelsea Technologies Group Ltd, UK). We maintained the downcast at a low velocity (<0.4 m s^−1^) and parked every 10 or 25 m to make a 30-s measurement. The fluorometer was programmed to carry out an FRRf acquisition sequence with 100 single-turnover flashes at 2-μs intervals and 40 low-energy flashes at 50-μs intervals in a total of 2.2 ms (Kolber et al., 1998); 36 acquisition sequences were averaged to increase the signal-to-noise ratio. In a single FRRf measurement, F_0_ is the initial fluorescence yield induced by a weak flash of light when the cells are in a dark-regulated state with all photosystem II (PSII, Table 2) reaction centers open for charge separation; after a series of brief excitation pulses, the fluorescence yield eventually reaches a maximal value (F_m_, Table 2), when the primary electron acceptors (Q_A_) are fully reduced and PSII reaction centers are all closed. The use of a very short flash time in FRRf minimizes the re-oxidation of Q_A_ (Kolber et al., 1998). By parameterizing the curve of increase of fluorescence yield from F_0_ to F_m_, the functional absorption cross section of PSII (σ_PSII_, Table 2) can be derived. The fluorescence yield is regulated by the competition between the processes of fluorescence (*F*), heat dissipation (*D*), and photochemistry (*P*); if *k* is the rate constant of these processes, *C* is the scale factor, *F*_0_ = *Ck*_*F*_/(*k*_*F*_ + *k*_*D*_ + *k*_*P*_), *F*_*m*_ = *Ck*_*F*_/(*k*_*F*_ + *k*_*D*_); then, *F*_*v*_/*F*_*m*_ = (*F*_*m*_ − *F*_0_)/*F*_*m*_ = *k*_*P*_/(*k*_*F*_ + *k*_*D*_ + *k*_*P*_), represents the maximum quantum yield of photochemistry. In a light-regulated state, *k*_*D*_ is assumed to change due to increased NPQ activity. Therefore, theoretically, $F_{0}^{\prime }=Ck_{F}/\left(k_{F}+xk_{D}+k_{P}\right)$, $F_{m}^{\prime }=Ck_{F}/\left(k_{F}+xk_{D}\right)$, and $F_{v}^{\prime }/F_{m}^{\prime }=k_{P}/\left(k_{F}+xk_{D}+k_{P}\right)$. In this study, in a light-regulated state, after a 2-s dark-adaptation, F_0_', F_m_', and σ_PSII_' were measured in the same way as F_0_, F_m_, and σ_PSII_. The drawback is that a 2-s dark-adaptation may cause a partial relaxation of NPQ that would overestimate F_0_' and underestimate F_v_'/ F_m_'. The decrease of *k*_*D*_ can be minimized by reducing the time in the dark, but a finite amount of time is required for Q_A_ to become adequately oxidized (Murchie and Lawson, 2013). The method of Oxborough and Baker (1997) can estimate F_v_'/F_m_' without the measurement of F_0_' but the measurements of F_0_ and F_m_ are required, such measurements could not be conducted *in-situ* during the daytime. We acknowledge the inherent limitation of *in-situ* or underway FRRf data, but this underestimation should not have affected the diel patterns of variable fluorescence that we observed in this study.

**Table 2:** Terms and parameters of active chlorophyll fluorescence technique used throughout the main text.

**Term**	**Definition**	**FastOcean APD chamber**
PSI	Photosystem I	
PSII	Photosystem II	
NPQ	Nonphotochemical quenching, including qI, qE and qT	
qI	Photo-inhibitory quenching, also called photoinactivation	
qE	Energy-dependent quenching, also called NPQf (rapidly reversible NPQ)	
qT	State-transition quenching	
n_PSII_	Ratio of PSII reaction centers to Chl*a*, mol PSII (mol Chl*a*)^−1^	
a_ph_	Phytoplankton absorption coefficient, m^−1^	
a_PSII_eflow_	Absorption coefficient for PSII electron flow (see main text), m^−1^	Dark
a_PSII_O2_	Absorption coefficient for oxygen flash yield (see main text), m^−1^	Dark
**In a dark-regulated state**		
F_0_	Initial fluorescence yield, unitless	Dark
F_m_	Maximum fluorescence yield, unitless	Dark
F_v_/F_m_	Maximum quantum yield of PSII photochemistry = (F_m_-F_0_)/F_m_, dimensionless	Dark
σ_PSII_	Functional absorption cross section of PSII, nm^2^ PSII^−1^	Dark
**In a light-regulated state when light is off for 2 s**		
F_0_'	Initial fluorescence yield, unitless	Dark
F_m_'	Maximum fluorescence yield, unitless	Dark
F_v_'/F_m_'	Maximum quantum yield of PSII photochemistry = (F_m_'-F_0_')/F_m_', dimensionless	Dark
σ_PSII_'	Functional absorption cross section of PSII, Å^2^ PSII^−1^	Dark
**Under ambient light**		
F'	Fluorescence yield under ambient light, unitless	Light
F_q_'/F_v_'	Proportion of open PSII under ambient light = (F_m_'-F')/(F_m_'- F_0_'), dimensionless	Light

The FastOcean APD fluorometer has two sensors. The “dark” sensor in a closed chamber measures the F_v_/F_m_ of samples at night and the ${\text{F}}_{\text{v}}^{\prime }$/${\text{F}}_{\text{m}}^{\prime }$ during the daytime. We used the term ${\text{F}}_{\text{v}}(\prime )$/${\text{F}}_{\text{m}}(\prime )$ to denote both F_v_/F_m_ and F_v_'/${\text{F}}_{\text{m}}^{\prime }$. Similarly, $\sigma_{\text{PSII}}(\prime )$ denoted both σ_PSII_ and $\sigma_{\text{PSII}}^{\prime }$. The “light” sensor with LED arrays facing upward is used to measure the F_q_'/F_v_' (Table 2). Although the fluorometer had different-color LED arrays, in this study we used only the blue light array based on the fact that the principal absorption band of chlorophyll *a* (Chl*a*) is in the blue region of the visible light spectrum. Therefore, a high vertical resolution was obtained by removing all the unnecessary measurements. Milli-Q water and seawater filtered through a 0.22-μm pore size polycarbonate filter (collected at the surface of station SEATS) were used to obtain the “baseline” fluorescence, which was used to correct subsequent ${\text{F}}_{0}(\prime )$ and ${\text{F}}_{\text{m}}(\prime )$ measurements. The correction was made once, and because the “baseline” fluorescence of the Milli-Q water and filtered seawater were very similar, the value of the Milli-Q water was used for correction. The “baseline” fluorescence accounted for 45–59% of the middy ${\text{F}}_{0}(\prime )$ at the surface, and the ${\text{F}}_{\text{v}}(\prime )$/${\text{F}}_{\text{m}}(\prime )$ values would have been lower by an average of 0.05 without the correction because ${\text{F}}_{0}(\prime )$ and ${\text{F}}_{\text{m}}(\prime )$ were generally low in the SCS basin. Thus, the “baseline” fluorescence correction was essential. However, Cullen and Davis (2003) found that the “baseline” fluorescence of filtered seawater was variable and lower than that of Milli-Q water. In such cases, use of an inappropriate “baseline” value based on Milli-Q water may lead to an overestimate of ${\text{F}}_{\text{v}}(\prime )$/${\text{F}}_{\text{m}}(\prime )$ and an underestimate of its variation.

### Carbon fixation measurements for net primary production (NPP) and photosynthesis-irradiance (P-I) curves

Both 24-h ^14^C uptake measurements to estimate NPP and 1-h P-I curve experiments were conducted (Table 1). NPP measurements at stations SEATS and SS1 were made with water collected in the morning from the surface and from depths corresponding to 70, 50, 23, 12, 3, 1, and 0.3% of surface PAR. Photosynthetically available radiation (PAR) sensors attached to the Seabird CTD and the FastOcean APD fluorometer were used to measure the vertical profiles of downwelling PAR, from which we estimated the vertical diffuse attenuation coefficient (K_d_) and then determined the sampling depths. NPP was measured in an incubator on deck. Sunlight was screened by different combinations of neutral density filters (LEE filters, UK) to simulate submarine irradiances. The seawater samples were placed in 60-mL polycarbonate bottles (two light bottles and one dark bottle for each depth) in a water bath, the temperature of which was maintained with seawater pumped from a depth of 5 m. The absence of a temperature gradient in the incubator was a drawback in the SCS basin, where the temperature difference between the surface and 100 m could be up to 10°C during the summer. Nevertheless, the impact of any error of NPP in the lower euphotic zone on the integration of NPP over the water column would be relatively small because the lower euphotic zone usually contributed substantially less than the upper euphotic zone to the integral of NPP. Carbon fixation was estimated from the uptake of NaH^14^CO_3_, which was added in trace amounts to the incubation bottles (Strickland and Parsons, 1972). After 24 h, the samples were filtered onto 25-mm–diameter glass fiber filters (Whatman, USA). The filters were processed immediately on board the research vessel. The radioactivity (CPM, counts per minute) on the filters was measured with a Tri-Carb 4810TR liquid scintillation counter after removing residual inorganic carbon by acid fuming overnight and immersing the filters in 4 mL of Ultima Gold scintillation cocktail (Perkin-Elmer, USA) until the filters became transparent. The type of filter is an issue in primary production measurements. GFF has the advantage of high flow rate and is a good choice for comparisons, but the high adsorption of dissolved organic carbon (DOC) is a problem, and the magnitude of adsorption process is unclear (Karl et al., 1998). Morán et al. (1999) have suggested that membrane filters are more suitable for measuring particulate primary production. We repeated radioactivity counting in the research vessel after 1 week (Karl et al., 1998). However, we found only a small increase in CPM values, unlike the large difference reported by Karl et al. (1998) in the North Pacific gyre. The release of ^14^C-DOC may be insignificant in the SCS basin. P-I curves were measured on samples from depths of 3, 8, 15, 76, and 102 m at station SS1 at both dawn and noon. The experiments were conducted with a photosynthetron with metal halide lamps (Xie et al., 2015). The seawater samples were placed in twelve 60-mL polycarbonate bottles in a water bath, the temperature of which was maintained by a cooler and heater and set to the surface temperature (~30°C) for the samples from 3, 8, and 15 m or to ~24°C for the samples from 76 and 102 m. After a 1-h incubation, the samples were filtered and processed following the procedures described above.

The NPP derived from 24-h incubations was calculated as follows:

(1) $$NPP=\frac{1.05\times DIC(DPM_{L}-DPM_{D})}{DPM_{tot}}$$

where the DPM (disintegrations per minute) was calculated from the CPM using the external standard and channel ratio method. The DPM_D_ (DPM in dark bottle) was subtracted from the DPM_L_ (DPM in light bottles) to eliminate the influence of adsorption and dark fixation of carbon. DPM_tot_ is the total activity of NaH^14^CO_3_ added to the incubation bottle. The activity of a small amount of NaH^14^CO_3_ mixed with 1 mL of CarboSorb-E was measured to estimate the value of DPM_tot_. DIC is the dissolved inorganic carbon concentration (g m^−3^), which was estimated from salinity using an empirical equation DIC = (9.9647+0.3944 × Salinity) applicable to SCS surface water (Xie et al., 2015). The factor of 1.05 was used to correct for isotope discrimination between ^14^C and ^12^C. Photosynthetic parameters were derived from the P-I curves by fitting to the equations (Platt et al., 1980):

(2) $$P^{B}=P_{s}^{B}\left[1-\exp\left(-\frac{\alpha I}{P_{s}^{B}}\right)\right]\exp\left(-\frac{\beta I}{P_{s}^{B}}\right)$$

(3) $$P_{m}^{B}=P_{s}^{B}\left[\frac{\alpha }{\alpha +\beta }\right]{\left[\frac{\beta }{\alpha +\beta }\right]}^{\frac{\beta }{\alpha }}$$

here *P*^*B*^ is the chlorophyll*-a*–normalized photosynthetic rate; $P_{m}^{B}$ is the light-saturated photosynthetic rate; α is the light-limited slope; and β is the photoinhibition parameter. In the absence of photoinhibition, β = 0, and the equation (2) became (Webb et al., 1974):

(4) $$P^{B}=P_{m}^{B}\left[1-\exp-\left(\frac{\alpha I}{P_{m}^{B}}\right)\right]$$

### Chlorophyll *a* (Chl*a*), pigments, and chemotaxonomic analysis

The Chl*a* and pigments for P-I curves and NPP were measured by the following procedure: 500 mL and 4 L of seawater were filtered onto two 25-mm–diameter glass fiber filters. The filters were then preserved in liquid nitrogen until analysis. The first filter was submerged in 90% acetone for Chl*a* extraction. After 16–24 h at −20°C in a dark environment, the Chl*a* was measured with a Trilogy fluorometer (Turner Designs, USA) by the method of Welschmeyer (1994). The second filter was submerged in *N,N*-dimethylformamide for pigment extraction and then mixed 1:1 (v:v) with 1-M ammonium acetate. The pigments in the extract with filter debris removed were measured in an UltiMate 3000 high-performance liquid chromatography system (ThermoFisher Scientific, USA). The pigments and their concentrations were determined by the retention times and peak areas with standard curve calibration. Chemotaxonomic analysis was carried out using CHEMTAX software (Mackey et al., 1996). This analysis gave the proportions and concentrations of nine phytoplankton classes (Dinoflagellates, Diatoms, Haptophytes_8, Haptophytes_6, Cryptophytes, Chlorophytes, Prasinophytes, *Synechococcus*, and *Prochlorococcus*) (Xiao et al., 2018). Based on the average, maximum and minimum values of pigment to Chl*a* ratio in the literature (Higgins et al., 2011), sixty matrices of seed ratios were randomly generated (as detailed in the Supplementary Material) for CHEMTAX analysis. The six best outputs with the lowest residuals were selected to calculate the average proportions and the standard deviations of the phytoplankton classes (Higgins et al., 2011). The results of many measurements were averaged to show the general patterns of phytoplankton community structure at stations SEATS and SS1 (*n* = 10 and *n* = 16, respectively). The propagation of error was made by Monte Carlo simulations. The proportion of each class was compared between the surface and DCM, and between stations SEATS and SS1, with two statistical methods. First, paired *t*-tests were used to give *p*-value. Then, we programmed a Monte Carlo simulation (*N* = 100,000 times) to compute the *t*-statistic with data randomly generated based on the mean value and the standard deviation given by CHEMTAX analysis. The number of rejections of the null hypothesis (H_0_) at the 0.05 significance level was counted, and the true significance level (${\hat{\alpha }}^{T}$) was defined by Albert (2009) and computed as:

(5) $${\hat{\alpha }}^{T}=\frac{\text{number of rejections of }{\text{H}}_{0}}{\text{N}}$$

We set the significance level of final results at both *p* < 0.05 and ${\hat{\alpha }}^{T}$ > 0.7.

### Estimates of “photosynthetic currencies” and absorption coefficients

In order to explore the eco-physiological cause of the low maximum quantum yield of carbon fixation of phytoplankton in the SCS basin, we estimated the “photosynthetic currency” and photosynthetic efficiency at station SS1. The term “photosynthetic currency” was proposed by Suggett et al. (2009a). The excitation of electrons by PSII and the fixation of CO_2_ by the dark reactions are different components of photosynthesis. The PSII-excited electrons (mmol e^−^) and net production of carbon (NPC, mmol C) are considered as different “currencies,” and there is an “exchange rate” between them. The rate of transfer of electrons from PSII is defined as the electron transfer rate (ETR), which is presumed to equal the rate of gross oxygen production (GOP, mmol O_2_) by the water-splitting reaction assuming that production of one oxygen molecule requires four electrons (Suggett et al., 2009a). The “exchange rate” or the electron requirement of NPC (mmol e^−^ (mmol C)^−1^) is the ratio between ETR and the rate of NPC, and the GOP:NPC ratio is estimated to be 25% of the “exchange rate”.

We roughly calculated the rates of four “currencies” at station SS1: (1) the daily-integrated ETR (daily–ETR, mmol e^−^ m^−2^ d^−1^) on 16 June. The ETR was calculated following Kolber and Falkowski (1993), and the daily–ETR was the sum of the hourly rates:

(6) $$\begin{array}{l} daily-ETR=\sum_{t=6}^{18}\sum_{z=0}^{100}E\left(z,t\right)\times corrected-\sigma_{PSII}^{\prime }\left(z,t\right)\\ \;\times Chla\left(z,t\right)\times \;n_{PSII}\times \frac{F_{q}^{\prime }}{F_{v}^{\prime }}\left(z,t\right)\\ \;\times \phi_{e}\times \left(\frac{\frac{F_{v}^{\prime }}{F_{m}^{\prime }}\left(z,t\right)}{0.65}\right)\times 24.3 \end{array}$$

where *E* was the incident PAR (mmol photons m^−2^ s^−1^); $\sigma_{PSII}\prime$ (Å^2^) was the functional absorption cross section of PSII spectrally corrected with Eequation (7), ${\bar{a}}_{ph}$ was the average phytoplankton absorption coefficient weighted to PAR and FRRf's excitation (Suggett et al., 2009a; Zhu et al., 2016). For example, ${\bar{a}}_{ph}\left(PAR\right)=\sum \left(Chla\times a_{ph}^{*}\left(\lambda \right)\times E\left(\lambda \right)\right)/\sum E\left(\lambda \right)$, the Chl*a*-specific phytoplankton absorption coefficient ($a_{ph}^{*}$) of Bricaud et al. (1995) was used to estimate ${\bar{a}}_{ph}$; Chl*a* (mg m^−3^) at each depth was interpolated linearly, and the molar mass of Chl*a* is 893.51 mg mmol^−1^; we assumed *n*_*PSII*_ to have a constant value of 0.002 mol PSII (mol Chl*a*)^−1^, but Kolber and Falkowski (1993) have suggested that its value is 0.0029 mol PSII (mol Chl*a*)^−1^ for a cyanobacteria-dominated phytoplankton assemblage, so we also did the calculation by assuming *n*_*PSII*_ of autotrophic eukaryotes and prokaryotes to be 0.002 and 0.003 mol (mol Chl*a*)^−1^, respectively. To estimate the *n*_*PSII*_, the phytoplankton community structure derived by CHEMTAX was used and interpolated linearly. ϕ_*e*_ is the actual quantum yield of electrons and was assumed to be one electron yielded from each PSII charge separation. The factor 24.3 was used to convert the combination of seconds to hours, Å^2^ to m^2^, mg m^−3^ to mmol PSII m^−3^, and mmol PSII to number of PSII.

(7) $$\text{corrected}-\sigma_{PSII}^{\prime }=(\frac{{\bar{a}}_{ph}\left(\text{PAR}\right)}{{\bar{a}}_{ph}\left(\text{FRRf}\right)})\times \sigma_{PSII}^{\prime }$$

(2) the daily–integrated ETR for oxygen flash yield (daily–ETR_O2_, mmol e^−^ m^−2^ d^−1^) on 16 June. Oxborough et al. (2012) introduced a K_R_ method to estimate the concentration of functional PSII reaction centers from *F*_0_, σ_*PSII*_, and the intensity of excitation light (*E*_*LED*_), K_R_ is an instrument-specific factor. Because the K_R_ method uses the oxygen flash yield as the standard rate for the correction curve, the calculated ETR may exclude electron flow through the Mehler reaction or PTOX pathway, and photorespiration. This method is independent of Chl*a* and *n*_*PSII*_. The daily–ETR_O2_ was calculated as:

(8) $$\begin{array}{l} daily-ETR_{O2}=\;\sum_{t=6}^{18}\sum_{z=0}^{100}E(z,t)\times (\frac{K_{R}\times F_{0}^{\prime }\left(z,t\right)}{E_{LED}\times \sigma_{PSII}^{\prime }(z,t)})\\ \;\times corrected-\sigma_{PSII}^{\prime }(z,t)\times \frac{F_{q}^{\prime }}{F_{v}^{\prime }}(z,t)\;\times 3600 \end{array}$$

where *K*_*R*_ was 40 × 10^18^ photons m^−3^ s^−1^, and *E*_*LED*_ was equal to 0.84 × 10^22^ photons m^−2^ s^−1^ in our FastOcean-APD system. The factor 3600 converts hours to seconds.

(3) the P-I model estimate of carbon fixation rate (mmol C m^−2^ d^−1^) during the day on 16 June. First, we estimated the hourly carbon fixation rate at 3, 8, 15, 76, and 102 m with the P-I model. Then, we integrated the values over time and depths using the trapezoid rule.

(9) $$\begin{array}{l} P-I-\text{modeled carbon fixation}=\sum_{t=6}^{18}\sum_{z=0}^{100}Chla(z,t)\times P_{s}^{B}\\ \times \left[1-\exp\left(-\frac{\alpha I(z,t)}{P_{s}^{B}}\right)\right]\\ \times \text{exp}\left(-\frac{\beta I(z,t)}{P_{s}^{B}}\right) \end{array}$$

where *I* was the incident PAR, the same as the PAR in equations (6) and (8), but with a different unit (μmol photons m^−2^ s^−1^), and the photosynthetic parameters were extrapolated by assuming they were linear functions of *I* at each depth.

(4) the rate of NPC (mmol C m^−2^ d^−1^) on 15 June was the NPP divided by 12 mg (mmol)^−1^.

Phytoplankton primary production has been modeled based on light absorption using the phytoplankton absorption coefficient (a_ph_, m^−1^) or Chl*a* as input (Lee et al., 2015). In order to examine the photosynthetic energetic stoichiometry from the beginning of total light absorption, we estimated the absorption coefficient for PSII electron flow (a_PSII_eflow_, m^−1^) and the absorption coefficient for oxygen flash yield (a_PSII_O2_, m^−1^) at 447 nm (the central wavelength of FastOcean blue LED excitation) as follows:

(10) $$\begin{array}{l} a_{PSII\_eflow}\;(447)=\sigma_{PSII}^{\prime }\times Chla\times n_{PSII}\\ \;\times \left(\frac{F_{v}^{\prime }}{F_{m}^{\prime }}/0.65\right)\times 0.00674 \end{array}$$

(11) $$a_{PSII\_O2}(447)=\frac{K_{R}\times F_{0}^{\prime }\left(z,t\right)}{E_{LED}}$$

where the parameters are the same as in Equations (6) and (8). The value of *n*_*PSII*_ was assumed to be 0.002 mol (mol Chl*a*)^−1^ for eukaryotic phytoplankton and 0.003 mol (mol Chl*a*)^−1^ for *Prochlorococcus* and *Synechoccocus*. The factor 0.00674 converts the combination of Å^2^ to m^2^, mg m^−3^ to mmol PSII m^−3^, and mmol PSII to number of PSII. We took the data in the upper 10 m at SS1 on 16 June for the average a_PSII_eflow_(447) and a_PSII_O2_(447). The shape of the spectrum mimiced that of Bricaud et al. (1995). Then we obtained the average a_ph_ (*n* = 14) in the SCS basin of about 0.10 mg Chl*a* (average surface Chl*a* concentration at SS1) at 5 m from a previous study (see the method in Xie et al., 2015), we also estimated a_ph_ of 0.10 mg Chl*a* using the empirical relationship of (Bricaud et al., 1995). The parameter a_ph_ was used to represent the total light absorption.

### Temperature, salinity, and nutrients measurements

The temperature and salinity of seawater were measured with a Seabird conductivity-temperature-depth (CTD) profiler, and the data were quality controlled and binned over 1-m depth intervals (Zhu et al., 2017). The mixed layer depth was calculated based on the potential density difference criterion (< 0.125 kg/m^3^). Temperature, salinity, and nutrients were ancillary data. Presenting part of these data was sufficient to characterize the environmental setting of the study. Temperature and salinity at depths of 0–125 m is reported in this study. Nutrients were analyzed onboard (Ocean Carbon Group of Xiamen University, Principal Investigator: Minhan Dai), but the data is unpublished for now and will be reported in another paper being prepared. Here, we briefly describe the vertical distribution patterns of nutrients: At the time of the NPP measurements at station SEATS and SS1, the nitrate+nitrite and phosphate were both below detection limits (the detection limits for nitrate+nitrite and phosphate were both 0.03 μmol L^−1^) in the upper 50 m; but on 16 June, when the P-I experiments were conducted, the nitrate+nitrite was depleted in the upper 75 m. Silicate was present in excess even at the surface (>1 μmol L^−1^). The nitracline with greatest nitrate+nitrite gradient was 75–100 m at station SEATS, and 100–125 m at station SS1. At the top of nitracline, the range of nitrate+nitrite concentration was about 0.8–3.0 μmol L^−1^, while it was 7.6–10.3 μmol L^−1^ at the bottom of nitracline.

## Results

### Temperature and salinity

**Figure 2:**
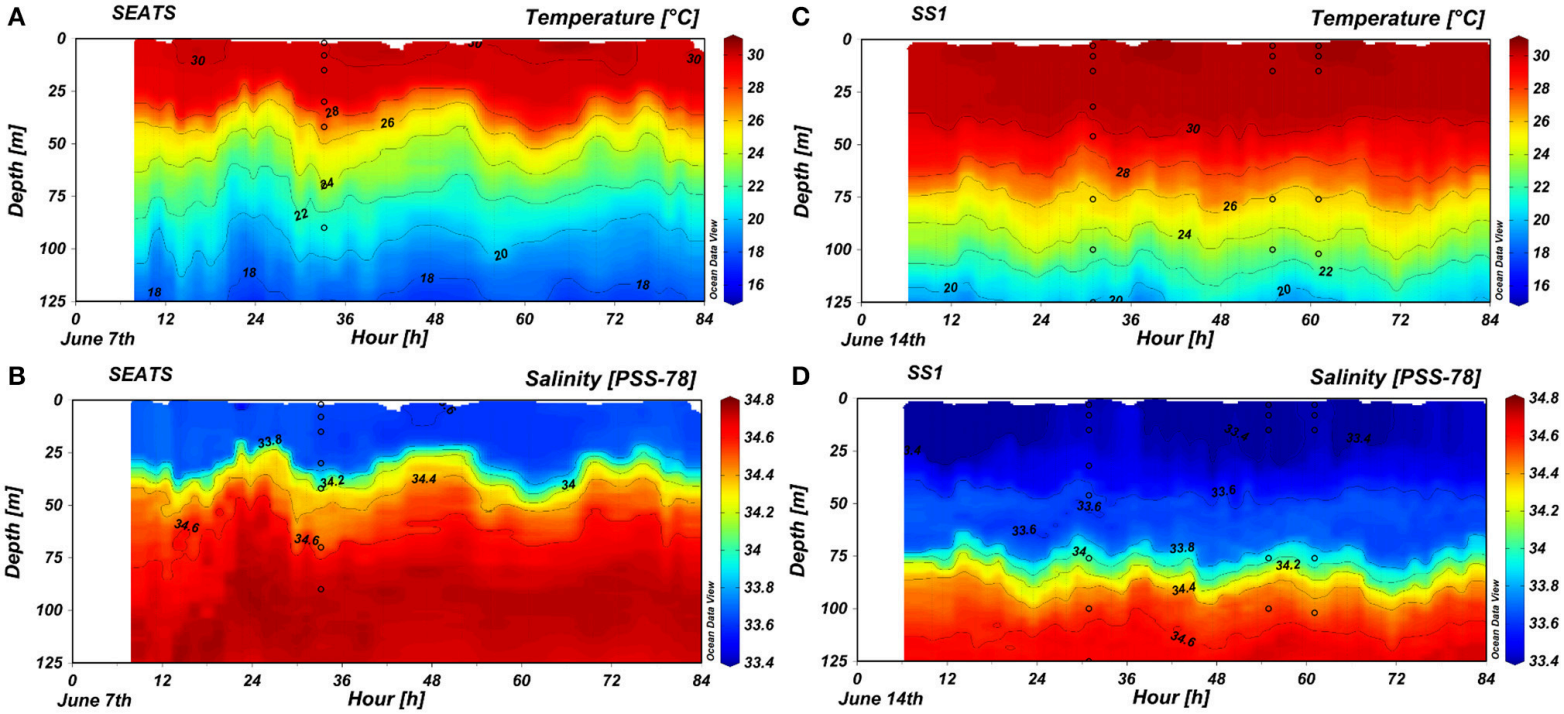
Changes in **(A,C)** temperature and **(B,D)** salinity over time in the upper 125 m at stations **(A,B)** SEATS and **(C,D)** SS1. The dots pinpointed the sampling depths for the net primary production measurements at SEATS on June 8th and at SS1 on June 15th, respectively; and for the photosynthesis-irradiance experiments at SS1 at 7:30 and 13:30 on June 16th, respectively.

The CTD profiles (Figure 2) showed a strong temperature gradient in the upper 125 m at both SEATS and SS1. The surface temperature underwent a diel cycle. The surface mixed layer was shallower at SEATS (average 24 m) than at SS1 (average 36 m). The temperature decreased greatly under the surface mixed layer. The average temperatures at 125 m were about 16.9° and 18.9°C at SEATS and SS1, respectively. The surface salinities at SEATS and SS1 were about 33.6 and 33.4, respectively. The fluctuations of the temperature and salinity isolines beneath the surface followed the tidal cycle; at SEATS the vertical excursions were greater than at SS1.

### Vertical and diel patterns of ${\text{F}}_{\text{v}}(\prime )$/${\text{F}}_{\text{m}}(\prime )$ and $\sigma_{\text{PSII}}(\prime )$

**Figure 3:**
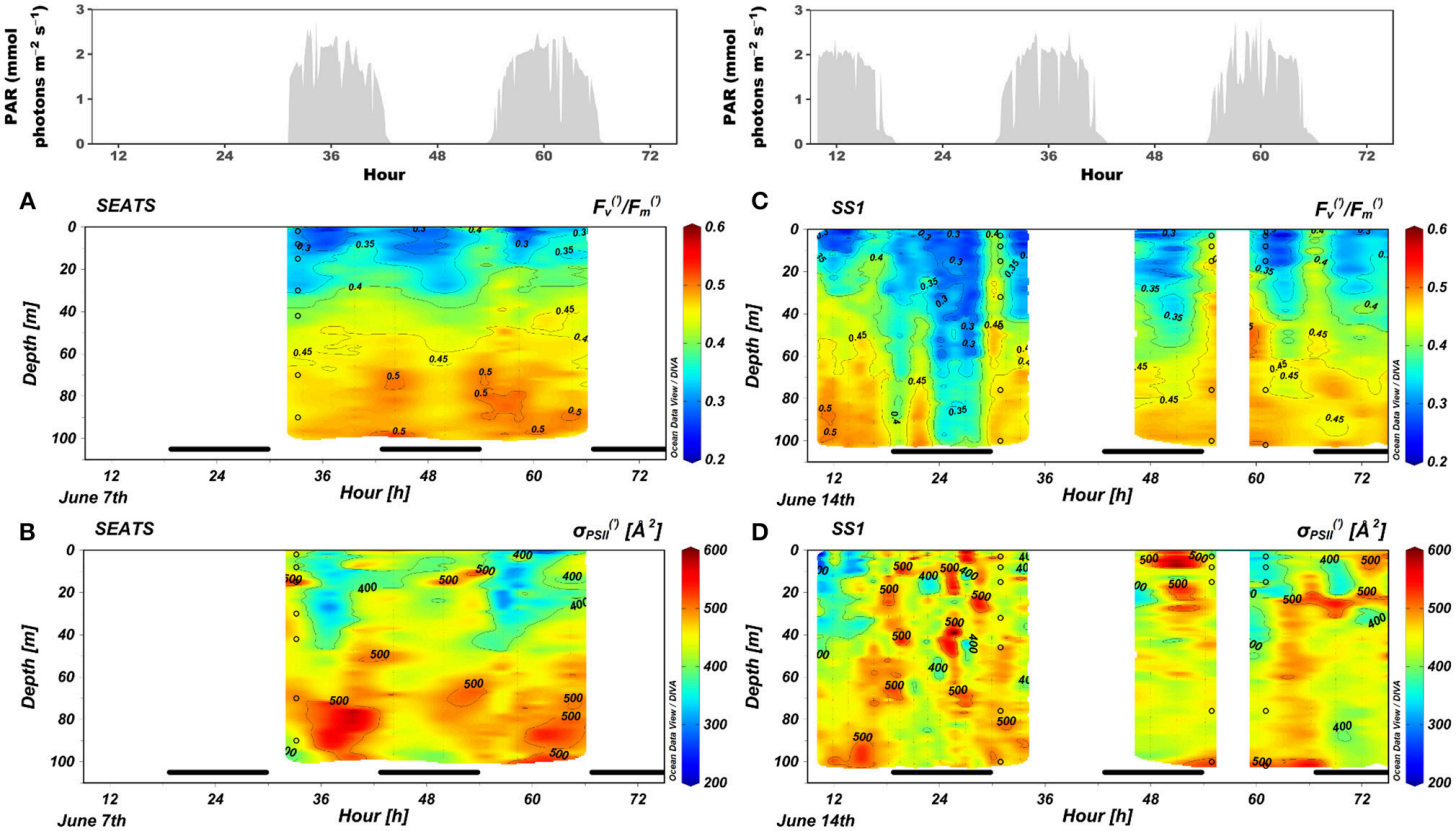
Changes in **(A,C)** ${\text{F}}_{\text{v}}(\prime )$/${\text{F}}_{\text{m}}(\prime )$ and **(B,D)** $\sigma_{\text{PSII}}(\prime )$ over time in the upper 100 m at stations **(A,B)** SEATS and **(C,D)** SS1. The upper panels were incident irradiance. The bars in the bottom of each panel indicated the night period. The dots are the same as those of Figure 2.

The FRRf measurements were conducted during sunny days (Figure 3). The ${\text{F}}_{\text{v}}(\prime )$/${\text{F}}_{\text{m}}(\prime )$ was low at the surface but increased with depth to values exceeding 0.45. The ${\text{F}}_{\text{v}}(\prime )$/${\text{F}}_{\text{m}}(\prime )$ in the mixed layer underwent a dramatic diel cycle characterized by a nocturnal decrease, a dawn maximum, and a decrease toward midday. The temporal variations of ${\text{F}}_{\text{v}}(\prime )$/${\text{F}}_{\text{m}}(\prime )$ between night and dawn were greater at SS1 than at SEATS; the nocturnal decrease at SS1 was severe between midnight and 04:30, when the 0.3 isoline descended to a depth as great as 60 m; otherwise, the nocturnal decrease diminished with increasing depths. The midday decreases of ${\text{F}}_{\text{v}}(\prime )$/${\text{F}}_{\text{m}}(\prime )$ were apparent only at depths shallower than 40 m, and a small increase of ${\text{F}}_{\text{v}}(\prime )$/${\text{F}}_{\text{m}}(\prime )$ at noon compared to the dawn value was observed in the DCM at SS1 (Figures 3A,C). Generally, $\sigma_{\text{PSII}}(\prime )$ was significantly depressed during midday in the surface layer. Values of $\sigma_{\text{PSII}}(\prime )$ ranged between 200 and 600 Å^2^ (Figures 3B,D).

### Vertical profiles of Chl*a* and NPP

**Figure 4:**
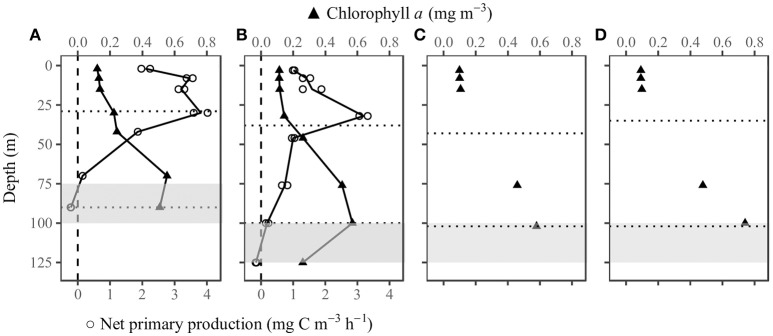
Vertical profiles of chlorophyll *a* (*n* = 1) and net primary production (*n* = 2) at stations **(A)** SEATS on June 8th and **(B)** SS1 on June 15th, and discrete chlorophyll *a* at **(C)** 7:30 and **(D)** 13:30 at station SS1 on June 16th. The upper dotted horizontal line indicates the mixed layer depth. The lower dotted horizontal line indicates the depth where PAR was 1% of the surface value. The gray area is the nitracline with the greatest nitrate+nitrite gradient. The integrated net primary production was 159.1 and 126.8 mg C m^−2^ d^−1^ at SEATS and SS1, respectively.

At the time when we collected the samples for NPP measurements, the surface Chl*a* was 0.12 mg m^−3^ at SEATS and 0.11 mg m^−3^ at SS1. The vertical Chl*a* profiles were Gaussian in shape. The Chl*a* changed only slightly within the mixed layer, but below the mixed layer there was a deep chlorophyll maximum layer (DCM), with a peak at about 70 m at SEATS and about 100 m at SS1. The maximum concentrations were both about 0.6 mg m^−3^ (Figures 4A,B; Supplementary Table 1). The column-integrated NPP was higher at SEATS than at SS1. At both stations, NPP was relatively high in the upper 30 m and decreased with increasing depth. The compensation depth where NPP equaled zero was between 70 and 90 m at SEATS and between 100 and 125 m at SS1. At both stations, NPP was depressed at the surface compared to greater depths within the mixed layer (Figures 4A,B; Supplementary Table 1). Although P-I experiments at SS1 were conducted 1 day after the NPP measurement, the Chl*a* concentrations had barely changed (Figures 4C,D).

### Phytoplankton community structure

**Figure 5:**
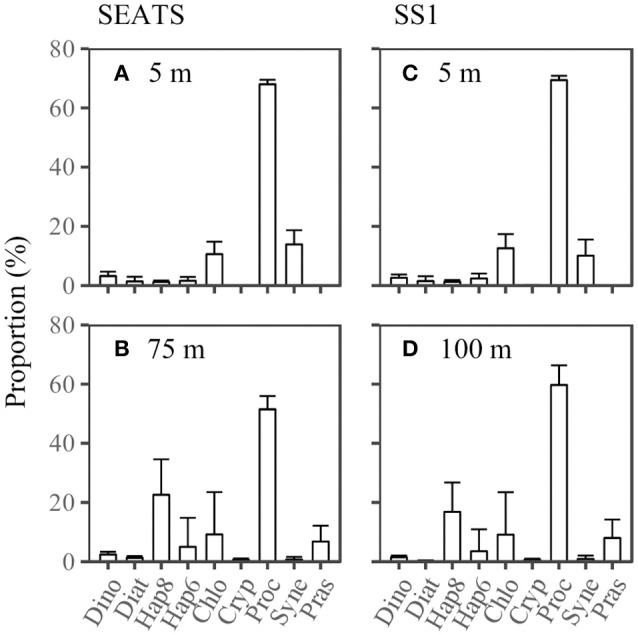
Phytoplankton community structure at **(A,C)** the surface and **(B,D)** the deep chlorophyll maximum at stations SEATS (*n* = 10) and SS1 (*n* = 16). The standard deviation is shown as the error bar. Dino, Diat, Hap8, Hap6, Chlo, Cryp, Proc, Syne, and Pras are the abbreviations for Dinoflagellates, Diatoms, Haptophytes_8, Haptophytes_6, Chlorophytes, Cryptophytes, *Synechococcus, Prochlorococcus* and Prasinophytes.

The proportions of phytoplankton classes in the community were averaged between 07:00 on 7 June and 07:50 on 8 June at station SEATS, and between 0:00 on 15 June and 24:00 on 16 June at station SS1. The results showed that the phytoplankton communities at SEATS and SS1 were both dominated by *Prochlorococcus*, followed by *Synechococcus* and Chlorophytes at the surface, and Haptophytes_8, Chlorophytes, Prasinophytes and Haptophytes_6 at the DCM. The proportions of Haptophytes_8 and Prasinophytes were much greater at the DCM than at the surface (*p* < 0.05, paired *t*-test; ${\hat{\alpha }}^{T}>$0.7, *t*-statistic with Monte Carlo simulation), whereas the proportion of *Synechococcus* decreased significantly (*p* < 0.05, paired *t*-test; ${\hat{\alpha }}^{T}>$0.7, *t*-statistic with Monte Carlo simulation). However, the community structure did not show any apparent difference between SEATS and SS1 (Figure 5).

### Photosynthetic parameters

**Figure 6:**
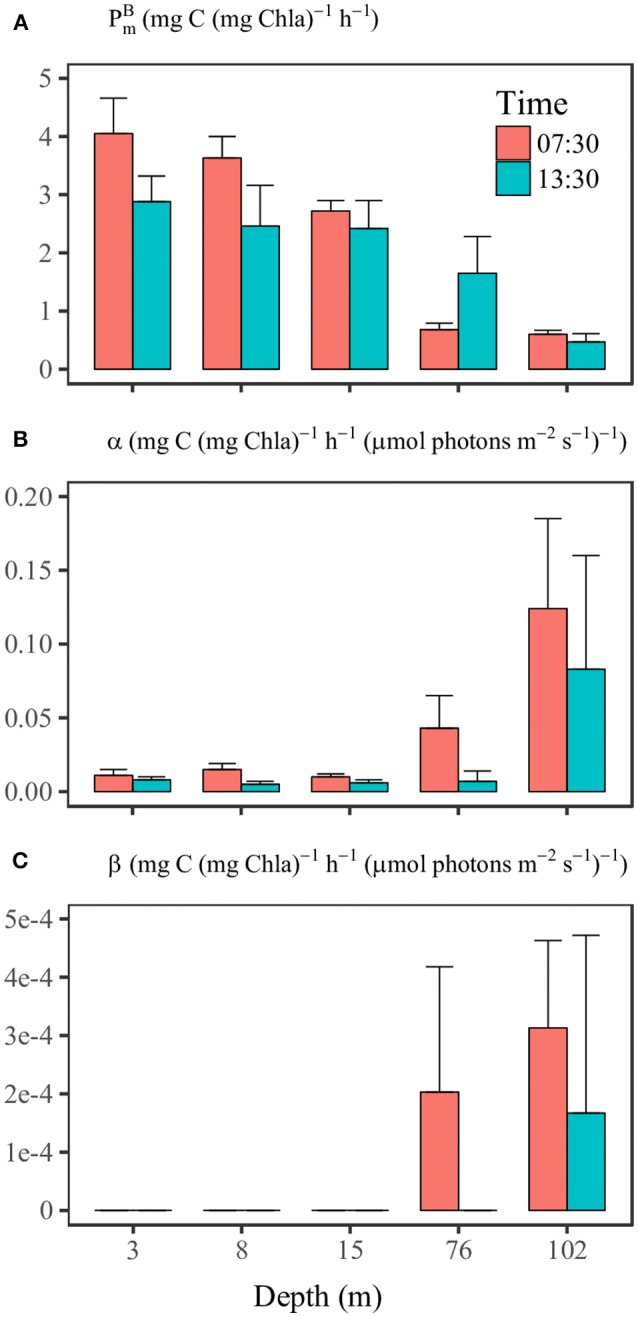
The comparisons of photosynthetic parameters **(A)** $P_{m}^{B}$, **(B)** α, and **(C)** β at different depths of station SS1 between 7:30 and 13:30 on June 16th. The 95% confidence level (*n* = 12) is shown as the error bar.

The results of the P-I experiment (Supplementary Figure 1 and Supplementary Table 2) at SS1 at 07:30 showed that the value of $P_{m}^{B}$ decreased with increasing depth from 4.05 mg C (mg Chl*a*)^−1^ h^−1^ to 0.60 mg C (mg Chl*a*)^−1^ h^−1^ at 102 m (Figure 6A). The initial slope α increased with depth from about 0.012 mg C h^−1^ (mg Chl*a*)^−1^ (μmol photons m^−2^ s^−1^)^−1^ at the surface to 0.124 mg C h^−1^ (mg Chl*a*)^−1^ (μmol photons m^−2^ s^−1^)^−1^ at 102 m (Figure 6B). The results of the P-I experiment at 13:30 indicated a significant temporal variation of photosynthetic parameters. Between 07:30 and 13:30, values of $P_{m}^{B}$ decreased from 4.05 to 2.88 mg C mg Chl*a*^−1^ h^−1^ at 3 m, from 3.63 to 2.46 mg C mg Chl*a*^−1^ h^−1^ at 8 m and from 2.72 to 2.42 mg C mg Chl*a*^−1^ h^−1^ at 15 m. There was less difference between $P_{m}^{B}$ values within the upper 15 m compared to the 07:30 experimental results (Figure 6A). In contrast, $P_{m}^{B}$ at 76 m was 0.68 mg C mg Chl*a*^−1^ h^−1^ at 07:30 but increased to 1.7 mg C mg Chl*a*^−1^ h^−1^ at 13:30, along with a substantial decrease of α (Figures 6A,B). The P-I curves showed apparent photoinhibition only at depths of 76 and 102 m (Figure 6C).

## Discussion

### Photosynthetic characteristics inferred from the diel patterns of ${\text{F}}_{\text{v}}(\prime )$/${\text{F}}_{\text{m}}(\prime )$ and photosynthetic parameters

The diel pattern of ${\text{F}}_{\text{v}}(\prime )$/${\text{F}}_{\text{m}}(\prime )$ at the surface in the SCS basin (Figure 3) was similar to the pattern that has been observed in the tropical Pacific Ocean (Behrenfeld and Kolber, 1999); there was a distinct nocturnal decrease and dawn maximum. Behrenfeld and Kolber (1999) attributed this pattern to nutrient stress induced by either macro-nutrient limitation or micro-nutrient limitation. NPQ could cause the decline in F_v_/F_m_ (Campbell and Tyystjärvi, 2012). It has long been thought that NPQ in cyanobacteria is primarily induced by state transitions (state-transition quenching, qT) which could happen in the dark (Campbell et al., 1998). Although the mechanism of state transitions in *Prochlorococcus* remains unclear (Partensky et al., 1999b), the mechanism may be similar: cyanobacteria use the same intermediates for both photosynthetic and respiratory electron flow; in the dark, respiration occupies the electron transport chain; the change in redox status of the chain induces state transitions, in this case, it is the reduced status that makes cells shift toward state 2, then the absorbed light is directed largely to PSI (Table 2), which increases the qT and lowers the quantum yield of PSII photochemistry (Campbell et al., 1998). The size of state transitions is affected very much by the nutritional status (Behrenfeld and Kolber, 1999). The abrupt increase of ${\text{F}}_{\text{v}}(\prime )$/${\text{F}}_{\text{m}}(\prime )$ at dawn is attributed to the recovery of PSI activity, which leads to oxidation of the electron transport chain. Cells then tend to be in state 1 (Campbell et al., 1998). Furthermore, our study showed generally less of a nocturnal decrease of ${\text{F}}_{\text{v}}(\prime )$/${\text{F}}_{\text{m}}(\prime )$ in the lower euphotic zone (Figure 3). The upper ocean shows strong stratification during the summer in the SCS basin (Figure 2). Nocturnal decreases of ${\text{F}}_{\text{v}}(\prime )$/${\text{F}}_{\text{m}}(\prime )$ were observed mainly within the surface mixed layer, which is nutrient depleted and where *Prochlorococcus* and *Synechococcus* dominated (Figure 5). Beneath the mixed layer, concentrations of both macro- and micro-nutrients increased near the bottom of the euphotic zone (Wu et al., 2003; Wen et al., 2006), where the proportion of photosynthetic eukaryotes increased greatly (Figure 5) and the LL ecotypes of *Prochlorococcus* may become dominant (Huang et al., 2012; Jing and Liu, 2012). No nocturnal decrease of ${\text{F}}_{\text{v}}(\prime )$/${\text{F}}_{\text{m}}(\prime )$ was detected at these depths (Figure 3).

The size of state transitions in cyanobacteria during the light period is small; therefore the qT may play little or no role in photo-protection from high-light inhibition (Campbell et al., 1998; Mullineaux and Emlyn-Jones, 2005). However, rapidly reversible nonphotochemical quenching (qE or NPQf) has been found in both HL and LL ecotypes of *Prochlorococcus*, although the mechanism is unclear (Bailey et al., 2005). Several proteins to perform qE have been found in *Synechococcus* (Scanlan et al., 2009). We may attribute the decrease of F_v_'/F_m_' in surface waters at noon (Figures 3A,C) to the existence of qE that is a photo-protection mechanism. Alternatively, because *Prochlorococcus* is characterized by a low repair rate of the PSII reaction center (Six et al., 2007; Murphy et al., 2017), the loss of functional PSII centers (photoinactivation or qI) in the high-light environment could be a more likely explanation. A decline of σ_PSII_' at noon was also observed (Figures 3B,D), although the relative change of σ_PSII_' was small compared to that of F_v_'/F_m_'. In higher plants and photosynthetic eukaryotes, a state 2 transition that moves mobile light-harvesting proteins from PSII to PSI could reduce the σ_PSII_ (Minagawa, 2011). However, this mechanism is not operative in cyanobacteria (Mullineaux and Emlyn-Jones, 2005). Campbell and Tyystjärvi (2012) have suggested that photoinactivation has little effect on σ_PSII_, but the study of Park et al. (1997) revealed that photoinactivation may cause a reduction of PSII antenna size.

The diadinoxanthin/diatoxanthin cycle and violaxanthin/zeaxanthin cycle are qE mechanisms carried out by diatoms and chlorophytes/prasinophytes, respectively. However, we did not find a clear pattern of either cycle for several reasons: first, the abundance of diatoms was low at the surface; second, we did not use dithiothreitol to inhibit the de-epoxidation reaction, which occurs rapidly (timescale of seconds to minutes) before filtering pigment samples (Bidigare et al., 2014); third, most zeaxanthin was contributed by *Synechococcus* and *Prochlorococcus*, in which there is no violaxanthin/zeaxanthin cycle.

**Figure 7:**
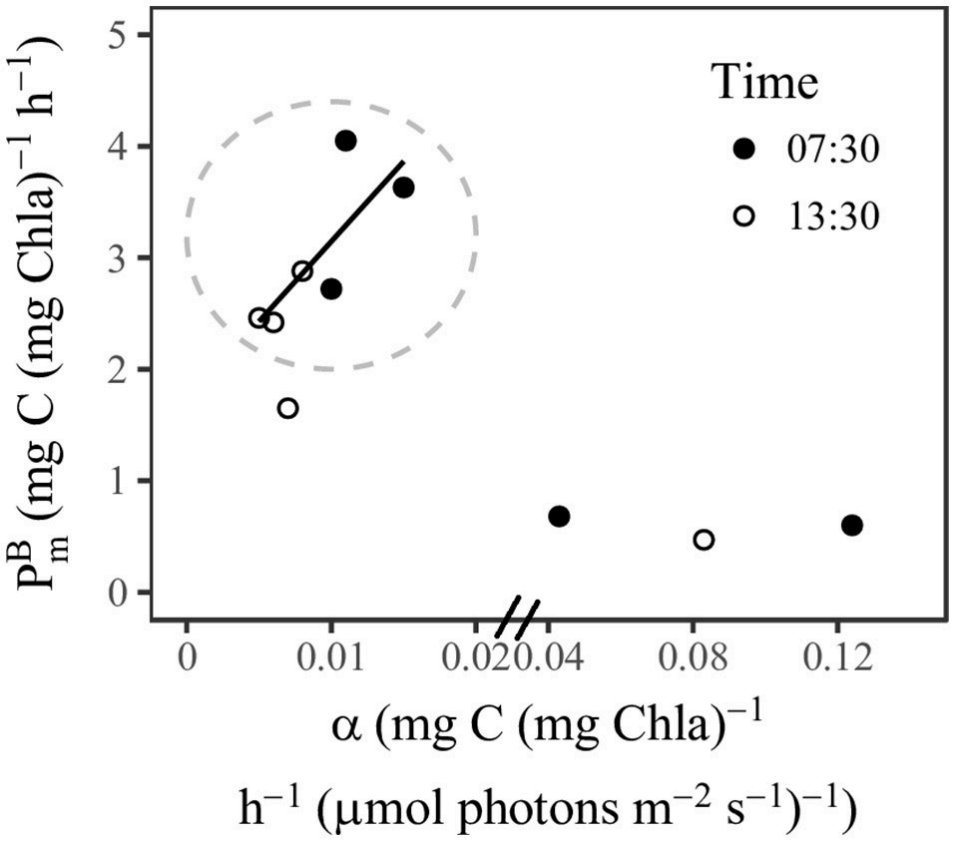
Scatter plot of P-I parameters $P_{m}^{B}$ against α at station SS1. Inside the circle were the samples within the surface mixed layer. The regression line indicated the concurrent change of $P_{m}^{B}$ and α (*r* = 0.79, *p* < 0.05).

$P_{m}^{B}$ and α measured at station SS1 were comparable with the values measured by Babin et al. (1996) in an oligotrophic area of the Atlantic Ocean. Both measurements revealed relatively low $P_{m}^{B}$ and α at the surface, and the α showed a dramatic increase with depth beneath the mixed layer. Compared with the compiled global values (Bouman et al., 2018), our surface $P_{m}^{B}$ was in the medium range, whereas the surface α was in the low range. The photosynthetic parameters also showed distinct diel variations, although we conducted P-I experiments only at dawn and noon (not at night). Previous studies have indicated that the photosynthetic parameters of phytoplankton usually go through nighttime minima (MacCaull and Platt, 1977; Harding et al., 1982). It is commonly accepted that the diel changes of photosynthetic parameters are under the control of cellular circadian cycles (Prézelin, 1992; Bruyant et al., 2005; Halsey et al., 2013). The highest rate of carbon fixation is associated with increased polysaccharide production during the S phase of the cell cycle (Halsey et al., 2013). The timing of the photosynthetic peak is dependent on the environment and could occur in morning (Harding et al., 1981; Bruyant et al., 2005; Schuback et al., 2016), noon, or afternoon (MacCaull and Platt, 1977; Harding et al., 1982). Experiments with *Prochlorococcus* (Bruyant et al., 2005) have shown results similar to those we observed from the *Prochlorococcus*-dominated communities in the SCS basin (Figures 3–6). $P_{m}^{B}$ and α peaked in the morning and then decreased at noon, along with declines of F_v_/F_m_ and σ_PSII_. Bruyant et al. (2005) have suggested that the diel changes of photosynthetic parameters are due not only to a cell cycle but more importantly to light regulation. The light utilization strategy of *Prochlorococcus* is to minimize their metabolic rate during high-light stress; then the repair rate of PSII reaction center is low, and the RuBiSCO transcription slows (Six et al., 2007; Mella-Flores et al., 2012; Murphy et al., 2017). This regulation may imply a light-dependence of RuBiSCO activity that essentially determines $P_{m}^{B}$. In this way the light and dark reactions of phytoplankton in oligotrophic environments respond coherently to high-light stress. The covariation of $P_{m}^{B}$ and α between the morning and noon within the surface mixed layer (Figure 7) suggests that the variations were “E_k_-independent”, a reflection of growth rate- or cell cycle-dependent changes in allocation of photosynthetic reductant (Behrenfeld et al., 2004; Halsey et al., 2013). However, in our study the change in σ_PSII_ may also contribute to the “E_k_-independent” variability by modifying α. At a depth of 76 m, the light at 07:30 might have been too low to activate PSI activity, and therefore photosynthetic capacity had not recovered from its nighttime minimum. At a depth of 100 m, the photosynthetic parameters barely changed over time, the implication being that there might have been a light threshold for phytoplankton photosynthesis to be fully functional.

### An indication of nutrient and iron stress in the central SCS basin

A previous study has shown that NPP is high in the winter and low in the summer in the SCS basin (Ning et al., 2004). During the summer, nutrients are depleted in the upper mixed layer, and the nutrient input from the deep layers is small. The growth of phytoplankton thus partially depends on nitrogen from atmospheric deposition and nitrogen fixation (Du et al., 2017). The macronutrient concentrations measured on this cruise were below the limit of detection in the upper 50 m. The diel patterns of ${\text{F}}_{\text{v}}(\prime )$/${\text{F}}_{\text{m}}(\prime )$ in our results are thus indicative of nutrient limitation in the SCS basin. Moreover, the magnitude of the diel changes of ${\text{F}}_{\text{v}}(\prime )$/${\text{F}}_{\text{m}}(\prime )$ was smaller at SEATS than at SS1. SS1 is at the center of the SCS basin, whereas SEATS is closer to the northern shelf. There has been no study that compares the nutrient supply rate between these two regions. A recent study has suggested that the influence of the Pearl River plume can reach the area of SEATS (He et al., 2016). Meanwhile, eddy activities are high in the northern SCS basin (Xiu et al., 2010). Allochthonous nutrient inputs from eddy pumping and river discharge could enhance the supply of nutrients for phytoplankton growth at SEATS, despite the low ambient nutrient concentrations. Our results also showed that SS1 had a deeper DCM and nitracline than SEATS (Figure 4), whereas SEATS had larger-amplitude internal waves than SS1 (Figure 2). The implication is that SEATS had a potentially higher upward influx of nutrient than SS1. The net result was that the NPP was higher at SEATS than at SS1. Interestingly, the differences in nutrient inputs and NPP did not result in different Chl*a* concentrations. In an oligotrophic ocean basin, the nutrient limitation may exert an influence on the species composition and photosynthetic performance of the phytoplankton (Xie et al., 2015), whereas the Chl*a* concentration is determined by the balance between bottom-up and top-down controls (Chen et al., 2009).

Only a few data are available to assess the iron concentrations at SEATS, but there is no information on iron concentrations in the central SCS basin. The iron concentrations in the upper mixed layer at SEATS in the summer are about 0.2–0.3 nM (Wu et al., 2003; Wen et al., 2006). Such low concentrations can induce seasonal iron limitation of phytoplankton (Sedwick et al., 2000). Wu et al. (2003) have suggested that iron may limit nitrogen fixation and inorganic phosphorus uptake in the SCS. However, the nutrient addition experiments conducted at SEATS showed that the addition of iron alone did not increase phytoplankton biomass (Chen, 2005). Nevertheless, whether iron and nitrogen are co-limiting has been predicted but not yet been determined (Browning et al., 2017). According to the diagnostic diagram of Behrenfeld et al. (2006, Figure 4), our results in the central SCS basin are projected to the low macro-nutrients and iron-limited category with an intermediate nocturnal decrease and dawn maximum of ${\text{F}}_{\text{v}}(\prime )$/${\text{F}}_{\text{m}}(\prime )$. Nutrient addition experiments will be required to clarify the nutrient limitation issue.

### Photosynthetic currencies and photosynthetic efficiency in the central SCS basin

What is the magnitude and variability of the “exchange rate” between PSII-excited electrons and NPC? The overview of a global dataset found this “exchange rate” is highly variable in the environment, where it ranges from 1.15 to 54.2 and averages 10.9 mmol e^−^ (mmol C)^−1^ (Lawrenz et al., 2013). Some other studies have examined GOP by oxygen isotopes tracer techniques, and the GOP:NPC ratio has been found to be conserved (about 3.3:1) under nitrogen limitation and dynamic light conditions in laboratory experiments, (Halsey and Jones, 2015), although field data have shown that this ratio may be higher than 3.3:1 under the natural conditions (Juranek and Quay, 2013). An alternative electron sink through a plastoquinol terminal oxidase (PTOX) has been found in cyanobacteria (Bailey et al., 2008), and it may play an important role in regulating the photosynthetic energetic stoichiometry. Mackey et al. (2008) found that the light saturation point of the electron transport is much higher than that of CO_2_ fixation, thereby the PSII electron transport and CO_2_ fixation of phytoplankton assemblages in the open ocean may be partially decoupled.

**Figure 8:**
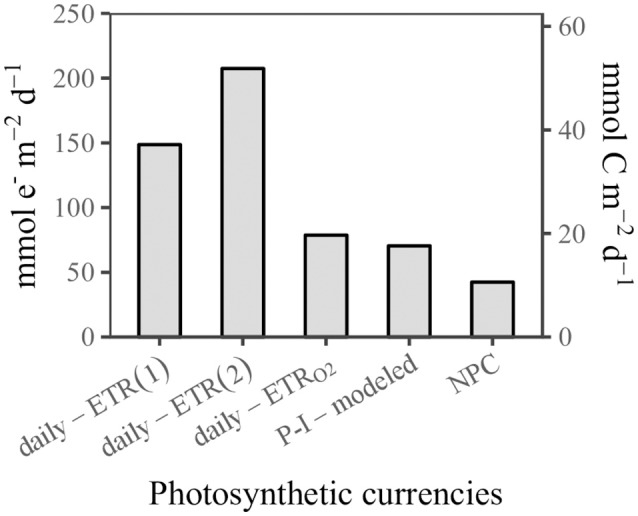
Comparison of different “photosynthetic currencies” (see the main text) at station SS1.

Figure 8 shows the “photosynthetic currencies” at SS1. The daily–ETR_O2_ was comparable to the P-I–modeled carbon fixation rate, assuming that four electrons are required to evolve one molecule of oxygen. The NPC was notably lower than the P-I–modeled carbon fixation rate. Short-term experiments (e.g., the 1-h P-I experiments) may record temporarily fixed carbon that is later respired during long-term experiments (such as NPP measurements) (Halsey et al., 2013). The time scales were different for the P-I–modeled carbon fixation and NPC. The latter excluded carbon respired at night. When the *n*_*PSII*_ was assumed to have a constant value of 0.002 mol PSII (mol Chl*a*)^−1^, the electron requirement of NPC (the ratio between daily–ETR and the rate of NPC) was about 14.0 mol e^−^ (mol C) ^−1^, and the derived GOP:NPC ratio was about 3.5:1, very close to the conserved ratio of 3.3:1 (Halsey and Jones, 2015). When the higher *n*_*PSII*_ of autotrophic prokaryotes was considered, the electron requirement of NPC was about 19.6 mol e^−^ (mol C) ^−1^, and the GOP:NPC ratio was about 4.9:1, which was similar to the ratio reported in the field (Juranek and Quay, 2013).

**Figure 9:**
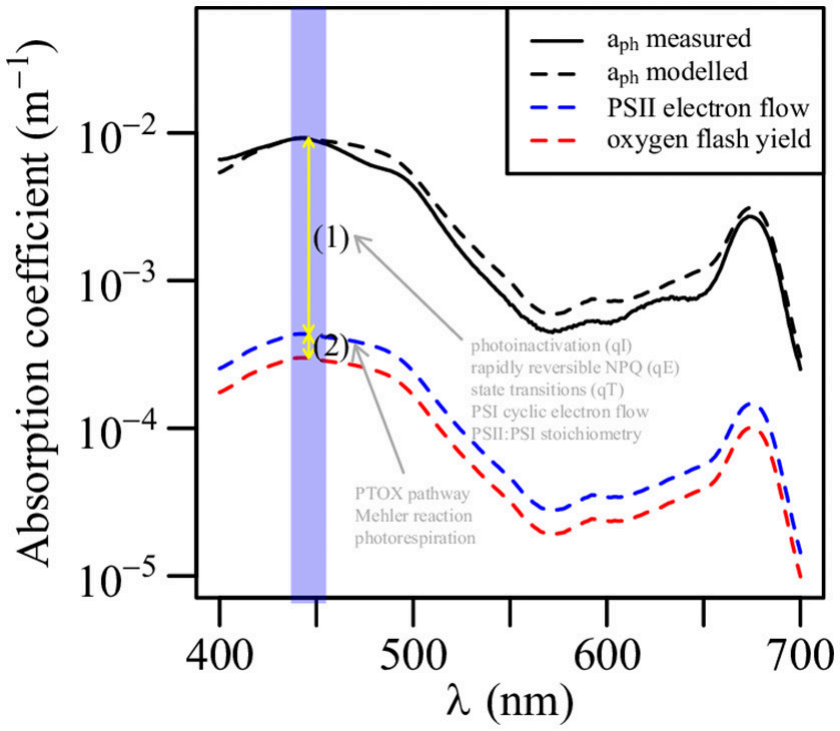
Comparison of phytoplankton absorption coefficient (a_ph_), absorption coefficient for PSII electron flow, and absorption coefficient for oxygen flash yield at the surface at station SS1 (see the main text). The blue band indicated the bandwidth of FastOcean blue LED.

Photosynthetic efficiency is traditionally described in terms of the conversion factor between NPP and the light absorbed by phytoplankton. The maximum quantum yield of carbon fixation ($\Phi_{m}^{C}$, mol C (mol photons)^−1^) is defined as the ratio between α (mg C (mg Chl*a*)^−1^ h^−1^ (μmol photons m^−2^ s^−1^)^−1^) and the Chl*a*-specific phytoplankton absorption coefficient [designated $a_{ph}^{*}$, m^2^ (mg Chl*a*)^−1^, (Bricaud et al., 1995)]. A previous study in the northern SCS has found that $\Phi_{m}^{C}\;$varies greatly across a nutrient gradient; the range is 0.003 to 0.104 mol C (mol photons)^−1^ (Xie et al., 2015). In the oligotrophic ocean, $\Phi_{m}^{C}$ is generally low (Cleveland et al., 1989; Babin et al., 1996; Hiscock et al., 2008; Xie et al., 2015). Figure 9 shows the four absorption coefficients we estimated. The modeled a_ph_ had a magnitude and shape similar to the measured a_ph_. The differences between the absorption coefficients represent the energetic stoichiometry during photosynthesis. Photoinactivation, qE, qT, and PSI cyclic electron flow may explain the difference between a_ph_(447) and a_PSII_eflow_(447); however, we have little understanding of these processes in *Prochlorococcus* in the field; based on the light utilization strategy of *Prochlorococcus*, photoinactivation may play an important role (Bruyant et al., 2005; Six et al., 2007; Mella-Flores et al., 2012; Murphy et al., 2017). The PTOX pathway, Mehler reaction, and photorespiration may account for the difference between a_PSII_eflow_(447) and a_PSII_O2_(447). The PTOX pathway has been well studied in *Prochlorococcus*, while the Mehler reaction is known to occur in *Synechoccocus* (Scanlan et al., 2009). Figure 9 shows a significant difference between a_ph_(447) and a_PSII_O2_(447), but most of the difference can be attributed to a_PSII_eflow_(447). The 1.5 orders of magnitude smaller a_PSII_O2_(447) than a_ph_(447) may explain the $\Phi_{m}^{C}$ as low as < 0.01 mol C (mol photons)^−1^ in the SCS basin that was reported in a previous study (Xie et al., 2015). The difference between a_PSII_eflow_(447) and a_PSII_O2_(447) accounted for some of the variability in the GOP:NPC ratio, a GOP:NPC ratio higher than 3.3:1 could be due to the presence of a PTOX pathway, the Mehler reaction, and photorespiration, which compete with carbon fixation for PSII-generated electrons but actually act as photoprotective mechanisms. The GOP:NPC ratio of 4.9:1 indicated a strong activity of these photoprotection processes, whereas the ratio of 3.5:1 may show relatively weak activity. An accurate value of *n*_*PSII*_ is therefore important to quantify the GOP:NPC ratio using the active chlorophyll fluorescence techniques.

## Concluding remarks

The diel pattern of phytoplankton photosynthesis in the picocyanobacteria *Prochlorococcus*–dominated SCS basin is typical of the pattern observed in the open ocean; it is characterized by a nocturnal decrease of ${\text{F}}_{\text{v}}(\prime )$/${\text{F}}_{\text{m}}(\prime )$ and midday depression of photosynthetic parameters in the nutrient-depleted surface layer. However, the fact that we found differences in the magnitude of the nocturnal decrease and dawn maximum of ${\text{F}}_{\text{v}}(\prime )$/${\text{F}}_{\text{m}}(\prime )$ between the SCS basin center and edge and the tropical Pacific Ocean indicates that the nutritional status of the phytoplankton assemblage may be variable across the basin. The light utilization strategy of *Prochlorococcus* helps to explain the photosynthetic efficiency in the oligotrophic oceans where they dominate. *Prochlorococcus* slows down metabolic activities like PSII repair and carbon fixation to cope with harmful high-light conditions. Estimates of “photosynthetic currencies” based on either constant or variable *n*_*PSII*_ resulted in GOP:NPC ratios that were close to previously reported values. This consistency may imply a common strategy of phytoplankton with respect to photosynthetic energetic stoichiometry (Halsey and Jones, 2015). On the other hand, primary production in the sea is estimated from phytoplankton absorption of light (Lee et al., 2015), but we found a large difference between the total light absorption and the absorption for PSII electron flow. The large extent of photoinactivation or NPQ in the oligotrophic oceans makes the direct use of the GOP:NPC ratio in primary production models difficult. However, these results could explain why $\Phi_{m}^{C}$ is typically very low in oligotrophic environments. Autonomous measurements by active fluorescence may in the future provide continuous estimates of primary production (Silsbe et al., 2015) and extend our ability to explore the ocean. This study has provided some insights into the photosynthetic characteristics of open-ocean phytoplankton. We also carried out baseline measurements that may provide a background for future research in this marginal sea.

## Author contributions

YX lead writer, designed and performed the experiments, analyzed and interpreted the data. EL data interpretation, scientific advice, language editing. LY junior graduate student, assisted to perform the experiments in the field, collected and analyzed the pigment samples; he is an early oceanographer budding into the scientific research using active fluorescence technology. BH program leader, scientific advice, designed the experiments.

### Conflict of interest statement

The authors declare that the research was conducted in the absence of any commercial or financial relationships that could be construed as a potential conflict of interest.
