# Supplementary material for: Diel Patterns of Variable Fluorescence and Carbon Fixation of Picocyanobacteria Prochlorococcus-Dominated Phytoplankton in the South China Sea Basin
Source: Front Microbiol. 2018 Aug 2;9:1589. doi: 10.3389/fmicb.2018.01589 (PMC6083051; doi:10.3389/fmicb.2018.01589)
Supplement: Supplementary file 1 [file Table_1.docx]

Supplementary Material

Diel patterns of variable fluorescence and carbon fixation of picocyanobacteria *Prochlorococcus*-dominated phytoplankton in the South China Sea basin

Yuyuan Xie, Edward A. Laws, Lei Yang, Bangqin Huang*

*** Correspondence:** Bangqin Huang: bqhuang@xmu.edu.cn

# Supplementary Method

This supplementary method is a guide to randomly generate 60 matrices of seed ratios for CHEMTAX analysis. **Avg** is the average seed ratio (supplementary Table 3), **Max** is the maximum seed ratio (supplementary Table 4), **Min** is the minimum seed ratio (supplementary Table 5), R is the random number between -1 and 1.

The matrix of seed ratios is randomly generated cell by cell with the equation $\boldsymbol{SR=Avg+D\times R}$, when R is positive, $\mathbf{D=Max-Avg}$, otherwise $\mathbf{D=Avg-Min}$.

This process is repeated until 60 matrices of seed ratios have been generated. In this study, this work was automatically done using R software.

# Supplementary Figures and Tables

## Supplementary Figures





**Supplementary Figure 1.** The P-I curves at different depths of station SS1 at (A-E) 7:30 and (F-J) 13:30 on June 16th, 2017.

## Supplementary Tables

**Supplementary Table 1.** The discrete chlorophyll *a* (Chl*a*, mg m^-3^) concentration and net primary production rate (NPP, mg C m^-3^ d^-1^) at stations SEATS and SS1 on June 8th and June 15th, 2017, respectively. NA means not applicable.

|  | **Depth (m)** | **Chl*a*** | **NPP (±standard error)** |
| --- | --- | --- | --- |
| **SEATS** | 2 | 0.12 | 2.10 (0.14) |
| June 8th, 2017 | 8 | 0.13 | 3.46 (0.10) |
|  | 15 | 0.14 | 3.21 (0.09) |
|  | 30 | 0.22 | 3.81 (0.22) |
|  | 42 | 0.24 | 1.86 (NA) |
|  | 70 | 0.55 | 0.15 (NA) |
|  | 90 | 0.51 | -0.21 (0.00) |
|  |  |  |  |
| **SS1** | 3 | 0.11 | 1.01 (0.03) |
| June 15th, 2017 | 8 | 0.11 | 1.41 (0.12) |
|  | 15 | 0.12 | 1.59 (0.29) |
|  | 32 | 0.14 | 3.19 (0.13) |
|  | 46 | 0.26 | 1.00 (0.04) |
|  | 76 | 0.51 | 0.74 (0.09) |
|  | 100 | 0.57 | 0.19 (0.04) |
|  | 125 | 0.26 | -0.16 (0.01) |

**Supplementary Table 2.** The P-I parameters with 95% confidence level (C.I.) at different depths of station SS1 at 7:30 and 13:30 on June 16th, 2017. NA means not applicable. The units are: $P_{s}^{B}$ [mg C (mg Chl*a*)^-1^ h^-1^], *α* [mg C (mg Chl*a*)^-1^ h^-1^ (μmol photons m^-2^ s^-1^)^-1^], *β* [mg C (mg Chl*a*)^-1^ h^-1^ (μmol photons m^-2^ s^-1^)^-1^], $P_{m}^{B}$ [mg C (mg Chl*a*)^-1^ h^-1^], $E_{k}$ [μmol photons m^-2^ s^-1^].

|  | **Chl*a*** | $\boldsymbol{P}_{\boldsymbol{s}}^{\boldsymbol{B}}$ | **95% C.I.** | ***α*** | **95% C.I.** | ***β* (10^-4^)** | **95% C.I.** | $\boldsymbol{P}_{\boldsymbol{m}}^{\boldsymbol{B}}$ | $\boldsymbol{E}_{\boldsymbol{k}}$ |
| --- | --- | --- | --- | --- | --- | --- | --- | --- | --- |
| **Time 7:30** |  |  |  |  |  |  |  |  |  |
| 3 m | 0.10 | 4.05 | 3.44–4.66 | 0.011 | 0.007–0.015 | NA | NA | 4.05 | 368 |
| 8 m | 0.10 | 3.63 | 3.26–4.00 | 0.015 | 0.011–0.019 | NA | NA | 3.63 | 242 |
| 15 m | 0.11 | 2.72 | 2.54–2.90 | 0.010 | 0.008–0.012 | NA | NA | 2.72 | 272 |
| 76 m | 0.46 | 0.70 | 0.61–0.79 | 0.043 | 0.021–0.065 | 2.03 | 0.99–4.18 | 0.68 | 16 |
| 102 m | 0.58 | 0.61 | 0.57–0.65 | 0.124 | 0.063–0.185 | 3.13 | 2.11–4.63 | 0.60 | 5 |
|  |  |  |  |  |  |  |  |  |  |
| **Time 13:30** |  |  |  |  |  |  |  |  |  |
| 3 m | 0.09 | 2.88 | 2.44–3.32 | 0.008 | 0.006–0.010 | NA | NA | 2.88 | 360 |
| 8 m | 0.09 | 2.46 | 1.76–3.16 | 0.005 | 0.003–0.007 | NA | NA | 2.46 | 492 |
| 15 m | 0.10 | 2.42 | 1.94–2.90 | 0.006 | 0.004–0.008 | NA | NA | 2.42 | 403 |
| 76 m | 0.48 | 1.65 | 1.02–2.28 | 0.007 | 0.000–0.014 | NA | NA | 1.65 | 235 |
| 100 m | 0.74 | 0.47 | 0.38–0.56 | 0.083 | 0.006–0.160 | 1.67 | 0.59–4.72 | 0.46 | 6 |

**Method to estimate 95% C.I.**

The 95% C.I. is used to show how accuracy of the mean (the value of parameters) is. It is calculated as:

$$C.I. =mean\pm(t \mathrm{multiplier}\times standard error)$$

Mean and standard error were estimated by P-I curve fitting (regression analysis), *t* multiplier is expressed as $t_{\alpha/2, df}$ and given by student *t* distribution function, while α here is the significance level, and df is the degree of freedom (number of observations minus number of parameters).

**Supplementary Table 3.** The average seed ratios for CHEMTAX analysis.

| Class / Pigment | Peridinin | 19-but-fucoxanthin | Fucoxanthin | 19-hex-fucoxanthin | Neoxanthin | Prasinoxanthin | Violaxathin | Alloxathin | Lutein | Zeaxanthin | Chlorophyll b | DV-chlorophyll a | Chlorophyll a |
| --- | --- | --- | --- | --- | --- | --- | --- | --- | --- | --- | --- | --- | --- |
| Dinoflagellates | 0.56 | 0 | 0 | 0 | 0 | 0 | 0 | 0 | 0 | 0 | 0 | 0 | 1 |
| Diatoms | 0 | 0 | 0.620 | 0 | 0 | 0 | 0 | 0 | 0 | 0 | 0 | 0 | 1 |
| Haptophytes_8 | 0 | 0.100 | 0.300 | 0.370 | 0 | 0 | 0 | 0 | 0 | 0 | 0 | 0 | 1 |
| Haptophytes_6 | 0 | 0.005 | 0.230 | 0.470 | 0 | 0 | 0 | 0 | 0 | 0 | 0 | 0 | 1 |
| Chlorophytes | 0 | 0 | 0 | 0 | 0.066 | 0 | 0.049 | 0 | 0.170 | 0.032 | 0.320 | 0 | 1 |
| Cryptophytes | 0 | 0 | 0 | 0 | 0 | 0 | 0 | 0.380 | 0 | 0 | 0 | 0 | 1 |
| *Prochlorococcus* | 0 | 0 | 0 | 0 | 0 | 0 | 0 | 0 | 0 | 0.390 | 0 | 1 | 0 |
| *Synechococcus* | 0 | 0 | 0 | 0 | 0 | 0 | 0 | 0 | 0 | 0.640 | 0 | 0 | 1 |
| Prasinophytes | 0 | 0 | 0 | 0 | 0.063 | 0.250 | 0.054 | 0 | 0.021 | 0.058 | 0.700 | 0 | 1 |

**Supplementary Table 4.** The maximum seed ratios for CHEMTAX analysis.

| Class / Pigment | Peridinin | 19-but-fucoxanthin | Fucoxanthin | 19-hex-fucoxanthin | Neoxanthin | Prasinoxanthin | Violaxathin | Alloxathin | Lutein | Zeaxanthin | Chlorophyll b | DV-chlorophyll a | Chlorophyll a |
| --- | --- | --- | --- | --- | --- | --- | --- | --- | --- | --- | --- | --- | --- |
| Dinoflagellates | 1.028 | 0 | 0 | 0 | 0 | 0 | 0 | 0 | 0 | 0 | 0 | 0 | 1 |
| Diatoms | 0 | 0 | 1.710 | 0 | 0 | 0 | 0 | 0 | 0 | 0 | 0 | 0 | 1 |
| Haptophytes_8 | 0 | 0.272 | 1.404 | 1.426 | 0 | 0 | 0 | 0 | 0 | 0 | 0 | 0 | 1 |
| Haptophytes_6 | 0 | 0.008 | 0.722 | 1.507 | 0 | 0 | 0 | 0 | 0 | 0 | 0 | 0 | 1 |
| Chlorophytes | 0 | 0 | 0 | 0 | 0.224 | 0 | 0.094 | 0 | 0.338 | 0.138 | 0.546 | 0 | 1 |
| Cryptophytes | 0 | 0 | 0 | 0 | 0 | 0 | 0 | 0.791 | 0 | 0 | 0 | 0 | 1 |
| *Prochlorococcus* | 0 | 0 | 0 | 0 | 0 | 0 | 0 | 0 | 0 | 1 | 0 | 1 | 0 |
| *Synechococcus* | 0 | 0 | 0 | 0 | 0 | 0 | 0 | 0 | 0 | 1.721 | 0 | 0 | 1 |
| Prasinophytes | 0 | 0 | 0 | 0 | 0.142 | 0.881 | 0.149 | 0 | 0.180 | 0.353 | 1.032 | 0 | 1 |

**Supplementary Table 5.** The minimum seed ratios for CHEMTAX analysis.

| Class / Pigment | Peridinin | 19-but-fucoxanthin | Fucoxanthin | 19-hex-fucoxanthin | Neoxanthin | Prasinoxanthin | Violaxathin | Alloxathin | Lutein | Zeaxanthin | Chlorophyll b | DV-chlorophyll a | Chlorophyll a |
| --- | --- | --- | --- | --- | --- | --- | --- | --- | --- | --- | --- | --- | --- |
| Dinoflagellates | 0.285 | 0 | 0 | 0 | 0 | 0 | 0 | 0 | 0 | 0 | 0 | 0 | 1 |
| Diatoms | 0 | 0 | 0.191 | 0 | 0 | 0 | 0 | 0 | 0 | 0 | 0 | 0 | 1 |
| Haptophytes_8 | 0 | 0 | 0.011 | 0 | 0 | 0 | 0 | 0 | 0 | 0 | 0 | 0 | 1 |
| Haptophytes_6 | 0 | 0 | 0.006 | 0.037 | 0 | 0 | 0 | 0 | 0 | 0 | 0 | 0 | 1 |
| Chlorophytes | 0 | 0 | 0 | 0 | 0.001 | 0 | 0.009 | 0 | 0.093 | 0.001 | 0.178 | 0 | 1 |
| Cryptophytes | 0 | 0 | 0 | 0 | 0 | 0 | 0 | 0.163 | 0 | 0 | 0 | 0 | 1 |
| *Prochlorococcus* | 0 | 0 | 0 | 0 | 0 | 0 | 0 | 0 | 0 | 0.050 | 0 | 1 | 0 |
| *Synechococcus* | 0 | 0 | 0 | 0 | 0 | 0 | 0 | 0 | 0 | 0.076 | 0 | 0 | 1 |
| Prasinophytes | 0 | 0 | 0 | 0 | 0.018 | 0.024 | 0.008 | 0 | 0.001 | 0 | 0.131 | 0 | 1 |
